# On Some Symptoms in Children Erroneously Attributed to Congestion of the Brain

**Published:** 1830-01-01

**Authors:** 


### OF SOME SYMPTOMS IN CHILDREN ERRONEOUSLY ATTRIBUTED TO CONGESTION OF THE BRAIN. By *Robert Gooch*, M.D.

THE above is the title of the sixth chapter in Dr. Gooch's recent publication, and the subject is of very great importance. The chapter opens in a ludicrous manner, considering the gravity of the subject; but the moral inculcated deserves to be remembered.

"I remember when a boy reading a story of two knights-errant who arrived on the opposite sides of a pedestal surmounted by a shield; one declared it was gold, the other that it was silver; growing angry, they proceeded to blows, and after a long fight each was thrown on the opposite side of the shield to that where he began the fight—when both immediately detected their error; the knight who had said it was silver finding that on the opposite side it was gold, and the knight who had said it was gold finding that on the opposite side it was silver. This story, a little modified, is a good illustration of the state of medical opinion in this age, perhaps in all ages; medical men have no occasion to tilt, for they all throng on one and the same side of the shield; they look only at the golden side, and never dream of the possibility that on the opposite side it may be of a different metal." 355.

Dr. G. properly remarks that two sets of symptoms are distinguishable in cases of disease, and require to be discriminated. One set of symptoms forms what may be called the physiognomy of the complaint—the other indicates the morbid state of organization on which the disease depends.

"Two patients complain occasionally of dimness of sight, swimming of the head, singing in the ears, and observe that if they turn the head on one side to look at an object they feel as if they should fall; but the one is plump, florid, and has a full pulse; the other is pale and thin, has cold hands and feet, and a pulse small and feeble. One practitioner bleeds them both; the other bleeds the one, but does all he can to give blood to the other. The latter cures both his patients; the former cures the one but ruins the health of the other; but such is the nature of the human mind that the cases *for* a preconceived opinion are retained easier than those *against* it. He remembers his good deed, forgets the other, or calls the case 'anomalous,' and marches on, without the slightest doubt that bleeding is the universal and sovereign remedy for dimness of sight, swimming of the head, and singing in the ears, save and except only in 'anomalous' cases." 357.

Dr. G. is anxious to call the attention of medical men to a disorder of children which he finds invariably attributed to, and treated as, CONGESTION or INFLAMMATION of the brain; but which he is convinced often depends on or is connected with, an opposite condition of the circulation. It is chiefly indicated by heaviness of head and drowsiness. The age of the little patients is generally from a few months to two years or three. They have, in our author's experience, been rather small of their age, in delicate health, and exposed to debilitating causes.

"The physician finds the child lying on its nurse's lap, unable or unwilling to raise its head, half asleep, one moment opening its eyes, and the next closing them again with a remarkable expression of languor. The tongue is slightly white, the skin is not hot, at times the nurse remarks that it is colder than natural; in some cases there is at times a slight and transient flush: the bowels I have always seen already disturbed by purgatives, so that I can scarcely say what they are when left to themselves; thus the state which I am describing is marked by heaviness of the head and drowsiness, without any signs of pain, great languour, and a total absence of all active febrile symptoms. The cases which I have seen have been invariably attributed to congestion of the brain, and the remedies employed have been leeches and cold lotions to the head, and purgatives, especially calomel. Under this treatment they have gradually become worse, the languor has increased, the deficiency of heat has become greater and more permanent, the pulse quicker and weaker, and at the end of a few days, or a week, or sometimes longer, the little patients have died with symptoms apparently of exhaustion. In two cases, however, I have seen, during the last few hours, symptoms of oppressed brain, as coma, stertorous breathing, and dilated and motionless pupil." 358.

Dr. Gooch relates an instructive case illustrative of the above remarks, where leeches were applied to a child two years old, and the result was fatal. This case and some incidental remarks are so interesting that we shall here insert them.

"A little girl, about two years old, small of her age and very delicate, was taken ill with the symptoms which I have above described. She lay dozing, languid, with a cool skin, and a pulse rather weak, but not much quicker than natural. She had no disposition to take nourishment. Her sister having died only a week before of an illness which began exactly in the same way, and which was treated by leeches and purgatives; and some doubts having been entertained by the medical attendant of the propriety of the treatment, leeches were withheld, but the child not being better at the end of two days, the parents, naturally anxious about their only surviving child, consulted another practitioner. The case was immediately decided to be one of cerebral congestion, and three leeches were ordered to be applied to the head. As the nurse was going to apply them, and during the absence of the medical attendants, a friend called in who had been educated for physic, but had never practised it, and who had great influence with the family: he saw the child, said that the doctors were not sufficiently active, and advised the number of the leeches to be doubled. Six, therefore, were applied; they bled copiously; but when the medical attendants assembled in the evening they found the aspect of the case totally altered, and that for the worse: the child was deadly pale, it had scarcely any pulse, its skin was cold, the pupils were dilated and motionless when light was allowed to fall on them, and when a watch was held to its eyes it seemed not to see; there was no squinting. Did this state of vision depend on the pressure of a fluid effused into the brain since the bleeding, and during this exhausted and feeble state of circulation, or did it depend on the circulation of the brain being too languid to support the sensibility of the retina? It is well known that large losses of blood enfeeble vision. I saw a striking instance of this in a lady who flooded to death. When I entered the chamber she had no pulse, and she was tossing about in that restless state which is so fatal a sign in these terrific cases. She could still speak, asked whether I was come, (she knew I had been sent for,) and said 'am I in any danger?—How dark the room is!—I can't see.' The shutters were open, the blind up, and the light from the window facing the bed fell strong on her face. I had the curiosity to lift the lid, and observe the state of the eye; the pupil was completely dilated, and perfectly motionless, though the light fell strong on it. Who can doubt that here the insensibility of the retina depended on the deficiency of its circulation? But to return to the little patient. The next day she had vomited her food several times; it was therefore directed that she should take no other nutriment than a dessert spoonful of ass's milk every hour, and this was strictly obeyed, and continued for several days. The child wasted, her features grew sharp, every now and then she looked fretful, and uttered a faint squeaking cry; the eye-balls became sunk in the socket, like those of a corpse that had been dead a month; the skin continued cool, and often cold, and the pulse weak, tremulous, and sometimes scarcely to be felt. Under this regimen, and in this way, she continued to go on for several days. At times she revived a little, so as to induce those who prescribed this treatment to believe confidently that she would recover, and she clearly regained her sight, for if a watch was held up to her she would follow it with her eyes. She lived longer than I expected; a full week, and then died with the symptoms of exhaustion, not with those of oppressed brain. The head was opened by a surgeon accustomed to anatomical examinations, and nothing was found but a little more serum than is usual in the ventricles." 361.

Our author concludes that the heaviness of the head and drowsiness, which were attributed to congestion in the brain, really depended on a deficiency of nervous energy—that the bleeding and scanty diet aggravated this state, "and insured the death of the child."

Dr. G. was summoned one day to see a child, to whose head the medical practitioner was about to apply leeches. He found the child lying on its nurse's lap, exactly in the state already described; with the same unwillingness to hold up its head—the same drowsiness—languor, absence of heat and of all symptoms of fever. Dr. G. took the medical attendant into another room, and told him the history of the preceding case and of several others of a similar kind. Instead of applying the leeches, they agreed to give from a pint and a half to a quart of ass's milk in the 24 hours—and to take ten minims of the aromatic spirit of ammonia every four hours. When they met next day, the nurse was walking about the nursery with the child upright in her arms. It looked happy and was laughing. The same plan was continued another day, and on the succeeding day Dr. Gooch took his leave.

"So inveterate is the disposition to attribute drowsiness in children to congestion of the brain, and to treat it so, that I have seen an infant, four months old, half dead from the diarrhœa produced by artificial food, and capable of being saved only by cordials, aromatics, and a breast of milk; but because it lay dozing on its nurse's lap two leeches had been put on the temples, and this by a practitioner of more than average sense and knowledge. I took off the leeches, stopped the bleeding of the bites, and attempted nothing but to restrain the diarrhœa, and get in plenty of nature's nutriment, and as I succeeded in this, the drowsiness went off, and the child revived. If it could have reasoned and spoken it would have told this practitioner how wrong he was; any one, who from long defect in the organs of nutrition, is reduced so that he has neither flesh on his body, nor blood in his veins, well knows what it is to lay down his head and doze away half the day without any congestion or inflammation of his brain. This error, although I have specified it only in a particular complaint of children, may be observed in our notions and treatment of other diseases, and at other periods of life. If a woman has a profuse hæmorrhage after delivery, she will probably have a distressing head-ache, with throbbing in the head, noises in the ears, a colourless complexion, and a quick, weak, often-thrilling pulse, all which symptoms are greatly increased by any exertion. I have seen this state treated in various ways, by small opiates, gentle aperients, and unstimulating nourishment, with no relief. I have seen blood taken away from the head, and it has afforded relief for a few hours, but then the head-ache, throbbing and noises have returned worse than ever; the truth is, that this is the acute state of what in a minor degree and in a more chronic form occurs in chlorosis, by which I mean pale-faced amenorrhœa, whether at puberty or in after-life. It may be called *acute chlorosis*, and like that disease is best cured by steel, given at first in small doses, gradually increased, merely obviating constipation by aloetic aperients." 365.

Dr. Gooch is unwilling to encumber this paper with a multiplicity of cases, but states generally that the above are only specimens of a class, of which he has seen enough to convince him that they deserve the attention of the profession. Dr. Gooch refers to the excellent remarks of Dr. Marshall Hall on this class of affections. Dr. G. candidly acknowledges that he has been anticipated by Dr. Hall, on this point, but so far from regretting it, he rejoices to find himself supported by such good authority.

"The children who were the subjects of this affection, and were thus treated, died not with symptoms of oppressed brain, but with those of exhaustion, and on examining the head after death, the blood vessels were unusually empty, and the fluid in the ventricles rather in excess ; in two instances death was preceded by symptoms of effusion, viz. blindness, a dilated pupil, coma, and convulsions ; and after death the ventricles were found distended with fluid to the amount of several ounces, the sinuses, and veins of the brain being remarkably empty. I believe the prevalent notion of the profession is, that all sudden effusions of water into the brain are the result of inflammatory action ; but putting aside for a moment this dogma of the schools, consider the circumstances of this case. For several days before death, all that part of the circulating system which was cognizable to the senses, was at the lowest ebb consistent with life, and after death the blood-vessels of the brain were found remarkably empty of blood, and the ventricles unusually full of water. From such facts I can draw no other inference than this, that this sudden effusion was a passive exudation from the exhalents of the ventricles occasioned by a state of the circulation the very opposite to congestion or inflammation. This is corroborated by the dissection of animals which have been bled to death. Drs. Saunders and Seeds, of Edinburgh, found that in animals bled to death, whether from veins or arteries, there was found more or less of serous effusion within the head, and Dr. Kelly thus expresses himself:—'If instead of bleeding usque ad mortem we were to bleed animals more sparingly and repeatedly, I have no doubt that we should succeed in draining the brain of a much larger quantity of its red blood ; but in such experiments we shall, I think, find a larger effusion of serum.' * * * 'Though we cannot, by general depletion, entirely or nearly empty the vascular system of the brain as we can the vessels of the other parts of the body, it is yet possible, by profuse hæmorrhages to drain it of a sensible portion of its red blood, that the place of this spoliation seems to be supplied both by extra and intra vascular serum, and that watery effusion within the head is a pretty constant concomitant or consequence of great sanguineous depletion.' But if this is true, it is of great practical importance, for if we take delicate feeble children, and by bleeding and purging for an imaginary congestion of the brain, reduce their circulation to a very low ebb and keep it so, we run the risk of producing that very effusion of serum into the brain which we are endeavouring by our remedies to prevent." 368.

Dr. Gooch does not expect that medical men will take his word as conclusive evidence for the truth of this paper—nor does he wish it. All he asks is that they will allow his observations and reasonings to lead them to look out for similar cases, and judge for themselves.

"With regard to the point that heaviness of head and drowsiness in children often depend not on congestion, but on deficiency of nervous power, and require for their cure not depletion, but support, I am quite satisfied that candid observers will find that I am right. With regard to the other point, that sudden effusion of serum may take place in the brain from a state of the circulation, the opposite to congestion or inflammation, it is more likely, even if true, to be overlooked; for such is the force of preconceived opinion, and such the prevalent notions on the subject, that the following will be the process in most minds. A child has been suffering some obscure symptoms for many days, when suddenly and unexpectedly it becomes blind, its pupils are dilated and motionless, it becomes convulsed, comatose, and dies. On opening the head serum is found in the ventricles, and without any further inquiry it is immediately taken for granted, that this effusion was the effect of overlooked inflammation of the brain, and regret is felt that active depletion had not been employed; the inference may be a correct one; all I contend for is, that it should not be taken for granted, but that those circumstances should be minutely inquired into which throw light on the state of the circulation in which the effusion occurred." 374.

We strongly recommend Dr. Gooch's observations to the attentive consideration of our professional brethren.

